# Early and Late-Term Follow-Up Results of Patients Diagnosed with Aortic Aneurysm or Aortic Dissection with Aortic Regurgitation Undergoing Aortic Valve Repair or Valve-Sparing Aortic Surgery

**DOI:** 10.21470/1678-9741-2020-0133

**Published:** 2021

**Authors:** Görkem Yiğit, Anıl Özen, Ferit Çetinkaya, Ertekin Utku Ünal, Hakki Zafer İşcan, Cemal Levent Birincioğlu, Ahmet Sarıtaş

**Affiliations:** 1 Department of Cardiovascular Surgery, Ankara City Hospital, Ankara, Turkey.

**Keywords:** Aortic Valve Insufficiency, Aortic Aneurysm, Aortic Valve, Heart Ventricles, Stroke Volume, Aneurysm, Dissecting, Postoperative Period, Replantation

## Abstract

**Introduction:**

Valve-reimplantation and remodelling techniques used in aortic reconstruction provide successful early, mid, and long-term results. We present our early and late-term experience with 110 patients with aortic regurgitation (AR) who underwent aortic valve repair (AVr) or valve-sparing aortic root surgeries (VSARS) due to aortic dissection or aortic aneurysm.

**Methods:**

Nine hundred eighty-two patients who underwent aneurysm or dissection surgery and aortic valve surgery between April 1997 and January 2017 were analysed using the patient database. A total of 110 patients with AR who underwent AVr or VSARS due to aortic dissection or aortic aneurysm were included in the study.

**Results:**

In the postoperative period, a decrease was observed in AR compared to the preoperative period (*P*<0.001); there was an increase in postoperative ejection fraction (EF) compared to the preoperative values (*P*<0.005) and a significant decrease in postoperative left ventricle diameters compared to the preoperative values (*P*<0.001). Kaplan-Meier analysis revealed one, two, four, and five-year freedom from moderate-severe AR as 95%, 91%, 87%, and 70%, respectively. Freedom from reoperation in one, two, and five years were 97.9%, 93.6%, and 81%, respectively. Eight patients (7.4%) underwent AVr during follow-up. Out of the remaining 100 patients, 13 (12%) had minimum AR, 52 (48%) had 1^st^-2^nd^ degree AR, and 35 (32%) had 2^nd^-3^rd^ degree AR during follow-up.

**Conclusion:**

For the purpose of maintaining the native valve tissue, preserving the EF and the left ventricular end-diastolic diameter, valve-sparing surgeries should be preferred for appropriate patients.

**Table:** 

Abbreviations, acronyms & symbols				
**AR**	**= Aortic regurgitation**		**INR**	**= International Normalization Ratio**
**ASCP**	**= Antegrade selective cerebral perfusion**		**LVEDD**	**= Left ventricular end-diastolic diameter**
**AVr**	**= Aortic valve repair**		**MFS**	**= Marfan syndrome**
**AVR**	**= Aortic valve replacement**		**NYHA**	**= New York Heart Association**
**BAV**	**= Bicuspid aortic valve**		**SCG**	**= Supracoronary graft replacement**
**CHF**	**= Chronic heart failure**		**SPSS**	**= Statistical Package for the Social Sciences**
**COPD**	**= Chronic obstructive pulmonary disease**		**STJ**	**= Sinotubular junction**
**CPB**	**= Cardiopulmonary bypass**		**VSARS**	**= Valve-sparing aortic root surgery**
**CVA**	**= Cerebrovascular accident**		**XCL**	**= X-clamp**
**EF**	**= Ejection fraction**			

## INTRODUCTION

Aortic valve reconstruction techniques have been available since the late 1950s. However, poor surgical outcomes resulted in aortic valve replacement (AVR) being predominantly preferred until the 1990s. In the early 1990s, aortic valve-sparing operations were initiated under the leadership of David and Yacoub and became widespread in the light of their long-term successful results^[1]^. Valve-reimplantation and remodelling techniques used in aortic reconstruction provide successful early, mid, and long-term results when applied to appropriate patients by an experienced surgical team.

Valve-protective surgery aims to preserve the patient's native valve and prevent prosthetic valve replacement surgery. In patients undergoing mechanical valve replacement, catastrophic complications such as valvular thrombosis and mechanical valve dysfunction due to ineffective coumadin use may occur. Furthermore, bleeding due to high International Normalization Ratio (INR) values and prosthetic valve endocarditis are amongst other significant complications. In addition, the possibility of life-threatening conditions and complications related to mechanical valve replacement, such as patient-prosthetic valve mismatch, pannus, and paravalvular leak, require the use of valve repair and valve-sparing surgery^[2]^.

The aim of our study was to determine early and late survival, degree of postoperative aortic regurgitation (AR), the incidence of redo cases, and early and late postoperative complication rates in patients diagnosed with aortic aneurysm or aortic dissection with AR undergoing aortic valve repair (AVr) or valve-sparing aortic root surgery (VSARS).

## METHODS

### Study Population

Nine hundred eighty-two patients who underwent aortic valve surgery and aortic aneurysm or dissection surgery between April 1997 and January 2017 were analysed using the patient database (Sarus and Avicenna automation systems) and examined by scanning files from hospital archives. A total of 110 patients with AR who underwent AVr or VSARS due to aortic dissection or aortic aneurysm were included in the study. Patients with AVR and patients without intervention of the aortic valve were excluded from the study.

There were only two mortalities in 110 patients (one intraoperatively and one at the fifth postoperative hour). Hence, a total of 108 patients were followed up. Preoperative data regarding age, sex, presence of Marfan syndrome (MFS), echocardiographic findings (aneurysm diameter, AR or stenosis degree, left ventricular end-diastolic diameter [LVEDD], ejection fraction [EF]), and data about other valve pathologies were obtained.

Intraoperative data regarding x-clamp (XCL) time, cardiopulmonary bypass (CPB) time, antegrade selective cerebral perfusion (ASCP) time, cooling degree, type of operation, need for inotropic support, and operative mortality data were collected.

In the postoperative period, early and late survival, mortality and morbidity rates, echocardiographic findings (AR or stenosis degree, LVEDDs, EF), incidence of being a redo case, causes of early and late mortality and morbidity, and postoperative complications were investigated. The minimum follow-up period was two months and the longest one was 108 months.

### Echocardiographic Assessment

All patients underwent post-repair intraoperative transepicardial or transoesophageal echocardiographic analysis. Transthoracic echocardiography was performed for all patients prior to discharge and at regular intervals for living patients with native valve during the course of follow-up.

### Statistical Analysis

All data were analysed using the Statistical Package for the Social Sciences (SPSS) software (SPSS Inc., Chicago, United States of America), version 15.0. The normal distribution of the variables was evaluated visually, using histograms and probability graphs, and analytically, using the Kolmogorov-Smirnov and Shapiro-Wilk tests. Normally distributed continuous variables were expressed in means and standard deviation whereas non-normally distributed continuous variables were presented using median and interquartile range values. Data on categorical variables were expressed in numbers and percentages. Preopereative and postoperative data was analysed using the Wilcoxon test. The Kaplan-Meier was used to evaluate freedom from medium-severe AR and freedom from reoperation. A different log-rank analysis was preferred to study the effect of valve-sparing surgery on survival. *P*-values < 0.05 were considered statistically significant.

## RESULTS

A total of 108 patients with AR who underwent AVr or VSARS due to aortic dissection or aortic aneurysm were included in the study. Twenty of these patients had aortic dissection (18.5%), seven (6.4%) had MFS, and five (4.6%) had bicuspid aortic valve (BAV) (Table 1).

**Table 1:** Preoperative patients' characteristics.

Sex (N=108)	Female	36 (33.3%)
	Male	72 (66.7%)
Age (years)		57.25±13.20 (20-82)
Hypertension (N=108)		72 (66.6%)
Diabetes mellitus (N=108)		12 (11.1%)
Hyperlipidemia (N=108)		15 (13.8%)
History of CVA (N=108)		6 (5.5%)
Chronic kidney disease (N=108)		1 (0.9%)
COPD (N=108)		5 (4.6%)
Marfan syndrome (N=108)		7 (6.4%)
CHF NYHA class (N=108)	Class I	37 (34.3%)
	Class II	34 (31.5%)
	Class III	28 (25.9%)
	Class IV	9 (8.3%)
Rhythm (N=108)	Sinus rhythm	106 (98.1%)
	Atrial fibrillation	2 (1.8%)
Operation type (N=108)	Urgent	20 (18.5%)
	Elective	88 (81.5%)
Patients' diagnosis (N=108)	Type A aortic dissection	20 (18.5%)
	Aortic regurgitation and ascending aortic aneurysm	84 (77.7%)
	Aortic regurgitation and ascending aortic aneurysm with arcus aortic aneurysm	4 (3.7%)
Bicuspid valve		5 (4.6%)
Operative procedure (N=108)	SCG + aortic valve intervention	94 (87%)
	Sinus remodelling + aortic valve intervention	14 (13%)
Additional surgical procedure performed on the aortic valve	Resuspension	74 (68.5%)
	Plication	25 (23.1%)
	Commissurotomy	9 (8.3%)
Additional surgical procedure (N=21)	Coronary artery bypass grafting	17 (15.7%)
	Mitral valve replacement	4 (3.7%)
	Total arcus replacement	10 (9.2%)

CHF=chronic heart failure; COPD=chronic obstructive pulmonary disease; CVA=cerebrovascular accident; NYHA=New York Heart Association; SCG=supracoronary graft replacement

The mean follow-up of our study was 25.29±24.81 (2-108) months. Seventy-two (66.7%) of the patients were male. There were only two mortalities out of 110 patients (one intraoperatively and one at the fifth postoperative hour). Hence, a total of 108 patients were followed up. Aortic valve intervention and supracoronary graft replacement (SCG) were performed in 94 patients (87%); aortic valve intervention and remodelling were performed in 14 patients (13%) (Table 1).

The mean age of the patients included in our study was 57.25±13.20 years. The youngest patient was a 24-year-old female with AR and ascending aortic aneurysm who underwent AVr and SCG. The oldest patient was an 82-year-old woman who underwent AVr, SCG, and two-vessel coronary bypass surgery for AR with coronary artery disease and ascending aortic aneurysm.

Mean operative time was 333.36±13.20 (180-780) minutes; mean CPB duration was 126.16±48.66 (54-352) minutes, and mean XCL duration was 82.28±32.24 (32-169) minutes. Only 42 patients (39.8%) underwent ASCP (Table 2).

**Table 2:** Intraoperative variables.

**Operation time (min)**		333.36±13.20 (180-780)
**XCL time (min)**		82.28±32.24 (32-169)
**CPB time (min)**		126.16±48.66 (54-352)
**ASCP time (min)**		20.98±13.60 (8-68)
**ASCP (min)**	Applied	42 (38.9%)
	Unapplied	66 (61.1%)
**Cooling degree (ºC)**		27.74±2.03 (19-34)

ASCP=antegrade selective cerebral perfusion; CPB=cardiopulmonary bypass; XCL=x-clamp

Postoperative data including the need for inotropic support, eryhrocyte suspension, and fresh frozen plasma are given in Table 3.

**Table 3:** Postoperative variables.

**Inotropic administration (N=108)**	None	37 (34.3%)
	Single	24 (22.2%)
	Two	47 (43.5%)
**Drainage (cc)**		910.93±726.67 (50-4250)
**Intubation time (h)**		18.62±41.04 (5-400)
**Erythrocyte suspension transfusion (Unit)**		1.42±2.30 (1-18)
**Fresh frozen plasma transfusion (Unit)**		3.34±5.56 (2-50)

In the postoperative period, 13 patients (12%) underwent revision for tamponade and six patients (5.6%) underwent revision for bleeding. Sixteen of these patients belonged to the SCG and AVr group, whereas three belonged to the remodelling and AVr group. Stroke developed in five patients (4.6%), pneumonia in one patient, mediastinitis in one patient (0.9%), and renal failure in one patient (0.9%). Eight patients (7.4%) underwent AVR during follow-up.

**Fig. 1:**
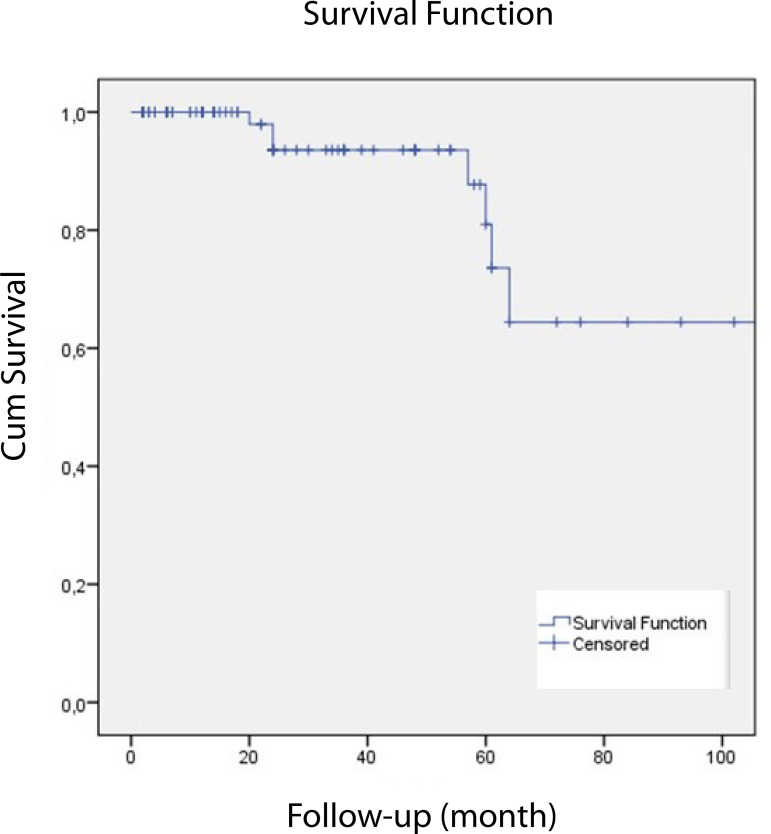
Kaplan-Meier curve for freedom from reoperation.

Eight patients (7.4%) underwent AVR during follow-up. One, two, and five-year freedom from reoperation were 97.9%, 93.6%, and 81%, respectively (Figure 1). Three patients who had undergone SCG and AVr with preoperative 2^nd^ degree and postoperative 3^rd^ degree of AR, underwent AVR at the 57^th^, 60^th^, and 64^th^ postoperative months; two patients who had undergone SCG and AVr with preoperative 3^rd^ to 4^th^ and 3^rd^ degree and postoperative 3^rd^ to 4^th^ degree of AR underwent AVR at the 20^th^ and 24^th^ postoperative months; one patient who had undergone Yacoub procedure with preoperative 1^st^ degree and postoperative 3^rd^ degree of AR underwent AVR at the 61^th^ postoperative month; and two patients who had undergone SCG and AVr due to acute aortic dissection with preoperative 1^st^ to 2^nd^ degree and postoperative 3^rd^ degree of AR underwent AVR at the 24^th^ and 108^th^ postoperative months (Table 4).

**Table 4:** Patients undergoing aortic valve replacement.

Patient	Operation type	Preop. AR	Postop. AR	Follow-up period (month)
1	SCG and aortic valve plication	2	3	57
2	SCG and aortic valve resuspension	2	3	60
3	SCG and aortic valve resuspension (bicuspid)	2	3	64
4	SCG and aortic valve resuspension	3/4	3/4	20
5	SCG and aortic valve resuspension	3	3/4	24
6	Aortic remodelling and aortic valve plication	1	3	61
7	SCG and aortic valve resuspension (type 1 dissection)	1/2	3	24
8	SCG and aortic valve resuspension (type 1 dissection)	1/2	3	108

AR=aortic regurgitation; SCG=supracoronary graft replacement

**Fig. 2:**
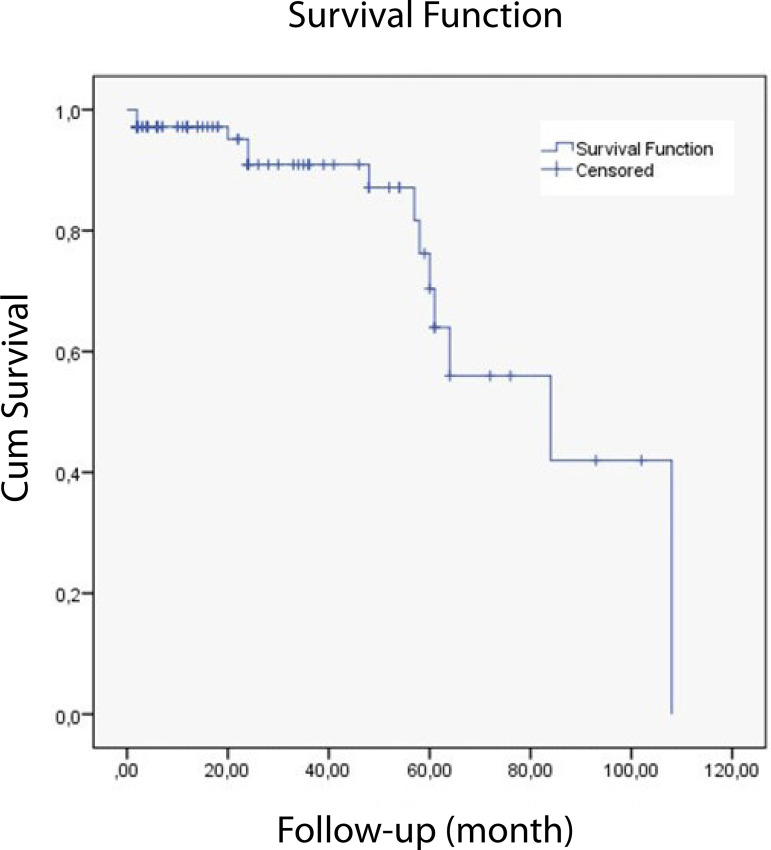
Kaplan-Meier curve for freedom from aortic regurgitation.

When the AR, EF, and diastolic ventricular diameters were compared in the preoperative and postoperative period, the differences were statistically significant. In the postoperative period, a decrease was observed in AR compared to the preoperative period (*P*<.001); there was an increase in postoperative EF compared to preoperative values (*P*<.005) and significant decrease in postoperative left ventricle diameters compared to preoperative values (*P*<.001) (Table 5). Kaplan-Meier analysis revealed one, two, four, and five-year freedom from moderate-severe AR as 95%, 91%, 87%, and 70%, respectively (Figure 2).

**Table 5:** Postoperative echocardiographic data.

	Preoperative	Postoperative	*P*-value
AR	2.05±0.61 (1-3.5)	1.41±0.78 (0-3)	<0.001
EF	52.71±8.01 (20-65)	54.45±7.49 (23-69)	<0.005
LVEDD	5.25±0.76 (4-8.2)	4.97±0.64 (3.9-7.4)	<0.001

AR=aortic regurgitation; EF=ejection fraction; LVEDD=left ventricular end-diastolic diameter

**Fig. 3:**
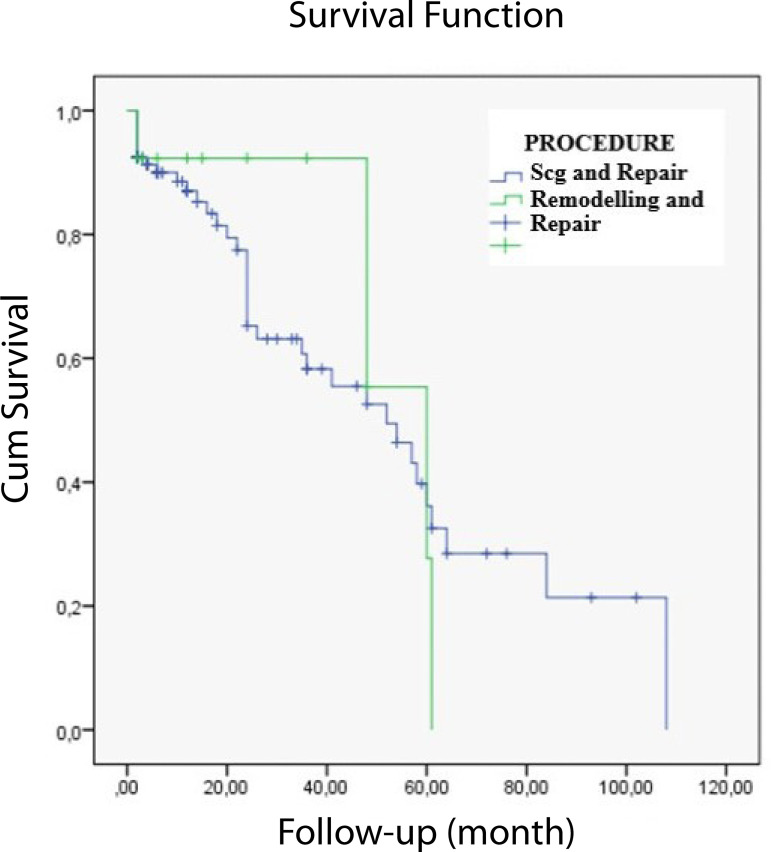
Kaplan-Meier comparison of aortic regurgitation in the remodelling and non-remodelling groups. SCG=supracoronary graft replacement

When the remodelling and non-remodelling groups were compared, no difference was found between the two methods in terms of freedom from AR (*P*=.832) (Figure 3).

Out of 100 remaining patients, 13 (12%) had minimum AR, 52 (48%) had 1^st^-2^nd^ degree AR, and 35 (32%) had 2^nd^-3^rd^ degree AR during follow-up.

## DISCUSSION

Aortic valve reconstruction techniques have been available since the late 1950s. However, poor surgical outcomes resulted in AVR being predominantly preferred until the 1990s. Valve-sparing operations gained accelaration after the 1990s due to high complication rates. Bleeding rates following mechanical valve replacement after 10 an 20 years are 16% and 61%, respectively^[3]^, and thromboembolic complication rates after 10 and 20 years were shown as 10% and 24%, respectively^[3]^. In addition, another major issue for this patient population is the economical burden caused by INR follow-up.

Bearing all the aforementioned disadvantages, interest in valve-sparing operations are growing. Benthall and its modifications have been accepted as golden standart^[1,4,5]^ for the surgical treatment of aortic root pathologies. Nevertheless, VSARS have become widely performed, due to successful long-term outcomes of these operations pioneered by David and Yacoub at the early 1990s^[1]^. Valve-sparing reimplantation and remodelling techniques used in aortic reconstruction possess successful short and long-term results, when applied to the appropriate patients by experienced surgical teams and should be considered primarily for patients with annuloaortic ectasia^[2]^. All the operations in this series were perfomed by several experienced surgeons and the early and long-term results are satisfactory.

There are few studies on isolated AVr in the literature. Freedom from reoperation rates were displayed as 95% in five years for AR resulting from prolapsus^[6,7]^. Aicher et al. shared their successful, long-term follow-up results of 15 years. They performed isolated AVr in 1083 patients between 1999 and 2015; 583 (54%) of these patients had tricuspid valves. Freedom from reintervention for tricuspid valves and bicuspid valves in five, 10, and 15 years were 94% and 84%, 81% and 90%, 78% and 71%, respectively^[6]^. This result reveals that success of the repair of tricuspid valves is higher than that of the bicuspid ones. Furthermore, presence of enlarged annulus or sinotubular junction (STJ) in their series was one of the main risk factors for failed valve repair. Combining annulus reduction or STJ remodelling with AVr have improved the long-term results^[8,9]^.

VSARS is the choice of treatment for aortic root disease with functional leaflets, even though AR accompanies the pathology. However, AR is not always due to root dilatation and may require a combined treatment using cusp repair. Significant preoperative AR and cusp repair seem to be risk factors for poor prognosis following VSARS^[10]^. Nonetheless, Schafers et al.^[11]^ applied an aggressive approach of combined leaflet prolapsus repair with VSARS and found no significance in operating times, mortality, and survival^[11,12]^. Besides this, Schafers preferred suture annuloplasy and Lansac used external ring annuluplasty in addition to reimplantation in order to stabilize the aortic root^[13]^.

In light of these developments, we retrospectively studied 108 patients, diagnosed with AR due to aortic aneurysm or dissection who underwent AVr or VSARS.

VSARS is usually preffered in young patients diagnosed with MFS and bicuspid aorta, aiming to stabilize the aortic annulus^[14-16]^. This study included 6.4% MFS patients and 4.6% congenital/bicuspid valve patients. The mean age of the study participants was 57 years and they can be considered as relatively young.

Preservation of cuspis geometry is the most important element of valve-sparing surgery. Together with minimal central AR, the final aim at the end of surgery should be no cuspis prolapsus and a coaptation height above the nadir of the aortic annulus.

There is a limited number of studies in the literature regarding the long-term outcome of patients undergoing VSARS. The ratios for survival after five and 10 years are 85-98.7% and 70-93.5%, respectively^[17-20]^. No mortalities occured in our series.

Acute aortic dissection is a significant risk factor for early mortality following VSARS^[18,21]^. Nevertheless, there were no mortalities amongst the patients who underwent surgery due to acute aortic dissection in our study. In contrast, the study of Shresta et al.^[18]^ revealed six early deaths (four patients with diagnosis of aortic dissection) out of 126 patients. The total number of patients undergoing surgery due to acute aortic dissection was 21 in their study. Twenty (18.5%) patients were operated with a diagnosis of acute type 1 aortic dissection in our study. All of these patients underwent SCG, AVR, and additional procedures. None of them underwent remodelling operations. There is no mortality during early, mid, and long-term follow-up of these patients. This may be explained by the fact that our patients with aortic dissection were not critically ill, malperfused, or comorbid patients as in the other mentioned studies.

Valve-sparing procedures are complex procedures requiring prolonged duration of XCL and CPB that may result in coagulopathies. Hence, the most common complication following such procedures is postoperative bleeding^[21]^. In the present study, following surgery, 13 (12%) patients developed tamponade and six (5.6%) patients underwent exploration due to bleeding. Five patients (4.6%) developed cerebrovascular accident, one (0.9%) had pneumonia, one (0.9%) had mediastinitis, and one (0.9%) had chronic renal failure.

The most significant problem after aortic valve-sparing and AVr procedures is AR and the need for consequent reoperation. Development of early AR following surgery is frequently due to technical failure^[22]^. None of the patients in the present study developed early AR. However, development of late AR mainly results from cusp degeneration and aortic root dilatation. David et al.^[23]^ announced five, 10, and 15-year freedom from mid-severe AR rates as 98.3%±3.5%, 92.9%±6.5%, and 89.4%±12%, respectively, in their study involving 296 patients. Furthermore, Coselli et al.^[24]^ shared their experience of 83 patients in 2014. They revealed two, four, six, and eight-year freedom from mid-severe AR rates as 94.8%±2.6%, 81.1%±5.3%, 77.8%±6%, and 73.9%±6.9%, respectively. The findings of the present study report the results of a maximum five-year period, evaluated by Kaplan-Meier analysis. Freedom from mid-severe AR rates are 95%, 91%, 87%, and 70% in one, two, four, and five years, respectively, and the result of freedom from AR in five years is similar to the one of Coselli et al.^[24]^

Eight patients in the present study underwent AVR during the follow-up period; one patient at the postoperative 20^th^ month and two patients at the postoperative 24^th^ month. Consequently, these three patients underwent AVR approximately at the postoperative second year. Four patients underwent AVR at the postoperative 57^th^, 60^th^, 61^st^, and 64^th^ months, which means that they underwent valve replacement approximately at the postoperative fifth year. Finally, one patient underwent AVR at the postoperative 108^th^ month, which is nine years following SCG and AVr due to acute type 1 aortic dissection. Annular stabilization was performed only in a single 73-year-old male patient, who had undergone AVr and remodelling due to AR and ascending aortic aneurysm. His preoperative echocardiography reported 1^st^ degree AR and postoperative echocardiography reported 3^rd^ degree AR. He underwent AVR at the postoperative 61^th^ month. None of the patients undergoing AVR had annular dilatation. According to the operation notes, the cause of valve replacement was cusp degeneration. The rate of freedom from reoperation in 10 years is 81-98% in the literature^[25]^. Leipzig group stated the five-year freedom from reoperation rate as 95.9%^[26]^. In addition, six patients out of 233 underwent AVR in the study of Kvitting et al.^[19]^ published in 2013. Their freedom from reoperation rates in five and 10 years were 98.0%±1.2% and 92.2%±3.6%, respectively. In another study, David et al.^[27]^ (2013) reported that seven patients underwent reoperation out of 374 patients. Freedom from reoperation in their report in 10, 15, and 20 years were 97.1%, 94.2%, and 94.2%, respectively. Our study revealed freedom from reoperation in one, two, and five years as 97.9%, 93.6%, and 81%, respectively. This result displays lower rates of freedom from reintervention compared to the aforementioned studies and maybe a result of presence of lower number of patients compared to other studies since our institute started performing valve-sparing operations 10 years later than the pioneering centers. Besides this, annular stabilization was applied only in a few patients and the operations were perfomed by six different surgeons.

Surgical approach for bicuspid valves is a topic much debated in the literature. Studies reveal worse outcomes for BAV compared to tricuspid ones, following valve-sparing operations^[28]^. A recent study by Shrestha et al.^[29]^ found high reoperation rate for patients with BAV (25%) with 7.2±4.7 years of follow-up. Their study revealed freedom from reoperation for patients with BAV as 68% in 10 years. However, Schafers et al.^[30]^ displayed freedom from reoperation in five years as 97%, for 173 patients undergoing AVr. Furthermore, the same surgical team published a more recent updated study including 316 patients demonstrating survival and freedom from reoperation rates in 10 years as 92% and 81%, respectively^[30,31]^. In our study, there were only five patients with BAV. Three of these patients underwent SCG and aortic valve resuspension, one underwent SCG and aortic valve commisurotomy, and one underwent Yacoub remodelling and aortic valve resuspension. A 43-year-old male patient with SCG and aortic valve resuspension underwent AVR at the 64^th^ postoperative month. The etiology of AR was cuspis prolapsus according to the operating note.

Another disputed subject is MFS. Martens et al.^[32]^ operated 104 patients with MFS by VSARS. They achieved 86% freedom from reoperation in 10 years and 80% freedom from reoperation in 20 years. David procedure is recommended instead of Yacoub procedure for patients with MFS, in order to provide annular stabilization and prevent annular dilatation^[33,34]^. In our study, there were seven patients with MFS. Only one patient underwent AVR at the 108^th^ postoperative month. This patient was a 48-year-old male who had undergone urgent surgery due to acute type 1 aortic dissection. Aortic valve resuspension and SCG replacement had been performed as an operation. The preoperative echocardiography revealed 1^st^-2^nd^ degree AR with an LVEDD of 5.8 cm. He became symptomatic with 3^rd^ degree of AR and an LVEDD of 7 cm at the postoperative nineth year and underwent AVR. Considering that his reoperation took place at the age of 57 years, a nine-year period without a mechanical valve and its disadvantages makes the decision to perform valve-sparing surgery during the first operation reasonable.

A significant finding of our study following aortic valve-sparing surgery, is the favorable values when comparing the preoperative and postoperative EF, LVEDD, and AR data. Monsefi et al.^[35]^ did not find any statistical difference between the preoperative and postoperative EF and LVEDD values. Nevertheless, there was an increase in postoperative EF compared to preoperative values and significant decrease in postoperative LVEDD compared to preoperative values in our study. Furthermore, in the postoperative period, a decrease was observed in AR compared to the preoperative period in the present study.

### Limitations

The retrospective nature of the study and shorter follow-up period compared to other large series are the main limitations of our study. Lower number of patients compared to other studies may be the main reason for no mortality and lower complication rates in the present study. Furthermore, application of annular stabilization in a limited number of patients is another limitation.

## CONCLUSION

AVr and valve-sparing procedures have become an alternative for valve replacement surgery for suitable patients in the last two decades. It is essential that these procedures have also started to appear in the current guidelines. Better understanding of the underlying pathology together with current advances in surgical techniques and long-term follow-up studies in the literature are going to allow for better results to be obtained. According to the European Society of Cardiology 2017 Guidelines, performing valve repair and valve-sparing procedures in young patients with aortic root dilatation is a class I indication. When we take a look at our last 20 years of experience in aortic valve-sparing procedures, there was no mortality during follow-up and the rates of freedom from reoperation in one, two, and five years were 97.9%, 93.6%, and 81%, respectively. In addition, freedom from mid-severe AR in five years was 70%. Only eight (7.4%) patients underwent AVR during follow-up. In conclusion, when considering the favourable postoperative echocardiographic findings, we believe that one should perform valve-sparing procedures for appropriate patients.

**Table:** 

Authors' roles & responsibilities	
GY	Substantial contributions to the conception or design of the work; or the acquisition, analysis, or interpretation of data for the work; drafting the work or revising it critically for important intellectual content; agreement to be accountable for all aspects of the work in ensuring that questions related to the accuracy or integrity of any part of the work are appropriately investigated and resolved; final approval of the version to be published
AÖ	Substantial contributions to the conception or design of the work; or the acquisition, analysis, or interpretation of data for the work; drafting the work or revising it critically for important intellectual content; agreement to be accountable for all aspects of the work in ensuring that questions related to the accuracy or integrity of any part of the work are appropriately investigated and resolved; final approval of the version to be published
FÇ	Substantial contributions to the conception or design of the work; or the acquisition, analysis, or interpretation of data for the work; drafting the work or revising it critically for important intellectual content; agreement to be accountable for all aspects of the work in ensuring that questions related to the accuracy or integrity of any part of the work are appropriately investigated and resolved; final approval of the version to be published
EUÜ	Substantial contributions to the conception or design of the work; or the acquisition, analysis, or interpretation of data for the work; drafting the work or revising it critically for important intellectual content; agreement to be accountable for all aspects of the work in ensuring that questions related to the accuracy or integrity of any part of the work are appropriately investigated and resolved; final approval of the version to be published
HZ	Substantial contributions to the conception or design of the work; or the acquisition, analysis, or interpretation of data for the work; drafting the work or revising it critically for important intellectual content; agreement to be accountable for all aspects of the work in ensuring that questions related to the accuracy or integrity of any part of the work are appropriately investigated and resolved; final approval of the version to be published
CLB	Substantial contributions to the conception or design of the work; or the acquisition, analysis, or interpretation of data for the work; drafting the work or revising it critically for important intellectual content; agreement to be accountable for all aspects of the work in ensuring that questions related to the accuracy or integrity of any part of the work are appropriately investigated and resolved; final approval of the version to be published
AS	Substantial contributions to the conception or design of the work; or the acquisition, analysis, or interpretation of data for the work; drafting the work or revising it critically for important intellectual content; agreement to be accountable for all aspects of the work in ensuring that questions related to the accuracy or integrity of any part of the work are appropriately investigated and resolved; final approval of the version to be published
